# The Improvement in Sleep Quality by Zizyphi Semen in Rodent Models Through GABAergic Transmission Regulation

**DOI:** 10.3390/nu16244266

**Published:** 2024-12-11

**Authors:** Mijin Kim, YuJaung Kim, Hyang Woon Lee, Kyung-Mi Kim, Singeun Kim, Seikwan Oh

**Affiliations:** 1 Department of Molecular Medicine, School of Medicine, Ewha Womans University, Seoul 07804, Republic of Korea; kimm2jin@gmail.com; 2 Department of Neurology, Ewha Medical Research Institute, Seoul 07804, Republic of Korea; yujaungkim@gmail.com (Y.K.); leeh@ewha.ac.kr (H.W.L.); 3 Department of Neurology, School of Medicine, Ewha Womans University, Seoul 07804, Republic of Korea; 4 Life Science Research Institute, NOVAREX Co., Ltd., Cheongju 28220, Republic of Korea; kkm3507@novarex.co.kr (K.-M.K.); kimsingun@novarex.co.kr (S.K.)

**Keywords:** sleep, pentobarbital, electroencephalogram, GABA_A_ receptor, jujuboside

## Abstract

**Background**: Sleep, a process physiologically vital for mental health, faces disruptions in various sleep disorders linked to metabolic and neurodegenerative risks. *Zizyphus* seed (Zizy) has long been recognized for its diverse pharmacological attributes, including analgesic, sedative, insomnia, and anxiety alleviation. **Objectives**: In this study, the sleep-prolonging effects of Zizy extract (100, 200 mg/kg), along with their characterizing compounds jujuboside A (JuA) (5, 10 mg/kg), were evaluated in a mouse model under a pentobarbital-induced sleep. Additionally, the efficacy of Zizy extract was examined on caffeine-induced insomnia in mice. **Methods**: To confirm the efficacy of Zizy extract on the structure and quality of sleep, an electroencephalogram (EEG) analysis of rats was performed using the MATLAB algorithm. Additionally, Western blot analysis and measurement of intracellular chloride influx were performed to confirm whether these effects acted through the gamma-aminobutyric acid (GABA)ergic system. Administration of Zizy extract showed no effect on the locomotor performance of mice, but the extract and their characteristic compounds significantly prolonged sleep duration in comparison to the pentobarbital alone group in the pentobarbital-induced sleep mouse model. Furthermore, this extract alleviated caffeine-induced insomnia in mice. **Results**: The administration of Zizy extract extended non-rapid eye movement sleep (NREMS) duration without inducing significant changes in the brain wave frequency. Zizy extract regulated the expression of GABA_A_ receptor subunits and GAD65/67 in specific brain regions (frontal cortex, hippocampus, and hypothalamus). JuA increased intracellular chloride influx in human SH-SY5Y cells, and it was reduced by GABA_A_ receptor antagonists. These results suggest that the sleep-maintaining effects of Zizy extract may entail GABAergic regulation. In summary, Zizy extract demonstrated sleep-prolonging properties, improved insomnia, and regulated sleep architecture through GABAergic system modulation. **Conclusions**: These findings suggest that Zizy extract has potential as a therapeutic agent for stress-related neuropsychiatric conditions such as insomnia.

## 1. Introduction

Sleep serves critical restorative, cognitive, and emotional functions [1,2]. The multifaceted significance of sleep lies in its pivotal role in memory consolidation, neural plasticity, and overall brain health [3,4,5,6]. Sleep deprivation detrimentally impacts attention, decision-making, and modulation of the immune system, thereby highlighting the importance of adequate and restful sleep [7,8,9]. Developing sleep aids from natural products is of profound interest due to their potential to mitigate the adverse effects associated with synthetic pharmaceuticals, such as tolerance and dependency [10]. Natural sleep aids, derived from botanical sources, possess a vast repertoire of bioactive compounds, including flavonoids and alkaloids, which exhibit promising hypnotic and anxiolytic properties [11].

Zizyphi Semen (Zizy) consists of dried ripe seeds from *Ziziphus jujuba* Miller var. *spinosa*, commonly utilized as both a functional food and a medicinal herb. Zizy, a traditional seed whose records of sedative and hypnotic properties span thousands of years, is believed to influence immune regulation and safeguard the blood–brain barrier [12,13]. This widespread utilization suggests it could serve as a functional food to counteract the onset of neurological disorders. A preliminary study showed that *Zizyphus* seed extract improved sleep duration [14]. Nevertheless, further systematic explorations are needed to fully elucidate the potential of *Zizyphus* seed as a safe and effective plant sleep aid. This study tested whether *Zizyphus* seed extract can increase sleep duration and improve sleep quality without disrupting sleep structure.

An electroencephalogram (EEG) analysis in rodents entails the precise monitoring and examination of electroencephalographic patterns, denoting neural oscillatory activity and synchrony in distinct brain regions. Through meticulous spectral analysis and coherence assessments, EEG signals can reliably delineate sleep–wake states in mice, including rapid eye movement (REM) and non-REM sleep, parallel to human sleep architecture [15,16]. These sophisticated neurophysiological techniques enable researchers to decipher sleep-related phenomena, such as sleep spindles and slow-wave oscillations, thereby providing valuable insights into sleep regulation and potential therapeutic interventions for sleep disorders in both mice and humans. Sleep quality is closely associated with NREM and REM delta and theta power. NREM slow-wave sleep (SWS) is marked by pronounced delta oscillations, indicating deep and restorative sleep. Theta activity during REM sleep aids in memory consolidation and dreaming. Balanced delta and theta power support optimal sleep quality, while disruptions may lead to sleep disorders and cognitive issues [17,18,19]. Polysomnographic studies of EEG power bands provide insights for sleep-related interventions and overall sleep health.

Thus, the objective of this study was to demonstrate that Zizy extract could extend and enhance the duration and quality of sleep and to elucidate the active mechanism by which Zizy improved sleep quality.

## 2. Materials and Methods

### 2.1. Chemicals

**Figure 1:**
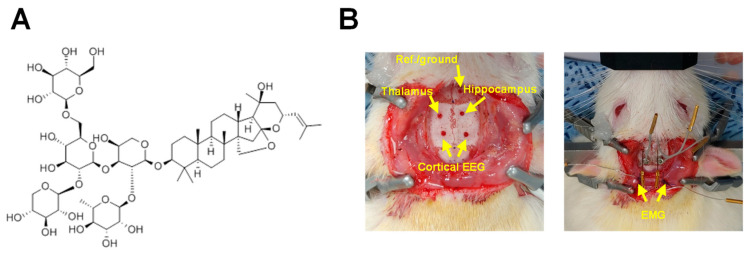
Chemical structure of jujuboside A compound (**A**) and schematic diagrams (**B**) of electrode placement for EEG/EMG recording and reference in the rat skull. The electrode implantation surgery was performed according to the following schematic. Electrodes and screws were inserted into the local field potential (LFP) of the left thalamus, LFP of the right hippocampus, reference/ground, bilateral cortical EEG, and two EMGs. EEG, electroencephalogram; EMG, electromyogram; LFP, local field potential.

Zizyphi Semen 30% fermented ethanol (Zizy) extract was obtained from NOVAREX (Cheongju, Republic of Korea). Jujuboside A (Figure 1A) was purchased from Sigma-Aldrich (St. Louis, MO, USA). Pentobarbital sodium was obtained from Hanlim Pharm (Seoul, Republic of Korea), and antibodies were purchased from Santa Cruz Biotechnology (Dallas, TX, USA) and Cell Signaling Technology (Danvers, MA, USA). N-(ethoxycarbonylmethyl)-6-methoxyquinolinium bromide (MQAE) was obtained from Thermo Fisher Scientific Inc. (Waltham, MA, USA). Ketoprofen was obtained from SCD Pharm. (Seoul, Republic of Korea), and lidocaine HCl injection 2%, sterile saline solution, and glucose injection (5%) were obtained from DAI HAN PHARM CO., Ltd. (Seoul, Republic of Korea). Chemicals not listed above were obtained from Sigma-Aldrich.

### 2.2. Animals

The animals, including male ICR mice (32–35 g) and Sprague Dawley (SD) rats (280–300 g), were procured from RaonBio (Yongin, Republic of Korea). To reduce the disruption of sleep and biorhythms due to gender differences, male animals were selected for the experiment. Animals were housed collectively (5 mice per cage; 2 rats per cage) in an environment maintained at 21–23 °C with a 12:12 h light–dark cycle and had unrestricted access to food and water. All procedures followed institutional guidelines and obtained approval from the Institutional Animal Care and Use Committee of Ewha Womans University School of Medicine (EWHA MEDIACUC past-040-4, 22-025).

### 2.3. Cell Culture

The SH-SY5Y cell line was purchased from the Korean Cell Line Bank (Seoul, Republic of Korea). The cells were cultured in a 5% CO_2_ incubator at 37 °C. The cells were grown in Dulbecco’s Modified Eagle Medium (DMEM; Welgene, Gyeongsan, Republic of Korea), containing 10% (*v*/*v*) fetal bovine serum (FBS) (Gibco, Karlsruhe, Germany), penicillin (10 U/mL) (Gibco), and streptomycin (10 µg/mL) (Gibco).

### 2.4. Pentobarbital-Induced Sleeping Test

Sleep evaluation was conducted by assessing the extension of sleep under pentobarbital induction. All experiments were conducted within the timeframe of 10:00 to 16:00, following previously established protocols [20,21], albeit with minor adjustments. This investigation utilized two distinct dosages of pentobarbital: a subhypnotic dose of 28 mg/kg and a hypnotic dose of 42 mg/kg. The ICR mice (*n* = 8–9) underwent a seven-day adaptation period and received treatments at 24 h intervals. They were subjected to a 24 h fasting period between successive administrations of the treatment agents, followed by a pentobarbital injection (i.p.) one hour after the last treatment. For Zizy extract administration, mice were segregated randomly into four groups for each pentobarbital dose (28 mg/kg and 42 mg/kg): pentobarbital only, and pentobarbital + Zizy at doses of 50 mg/kg, 100 mg/kg, and 200 mg/kg (p.o.). In the case of jujuboside A (JuA) treatment, mice were divided into three groups for each pentobarbital dose: pentobarbital only, and pentobarbital + JuA at doses of 5 mg/kg and 10 mg/kg (i.p.). Following the administration of pentobarbital, mice were individually placed in cages and monitored for sleep latency and duration measurements. Sleep latency was determined from the time of pentobarbital injection until the onset of sleep, while sleep duration was calculated as the difference in time between the loss and recovery of the righting reflex.

### 2.5. Caffeine-Induced Insomnia Model

The caffeine-induced insomnia mice were used in the pentobarbital-induced sleep model to assess the potential alleviation of insomnia. Zizy extract was orally administered twice at 24 h intervals, while caffeine (10 mg/kg) or saline was treated intraperitoneally 20 min after the last extract administration. The pentobarbital was administered intraperitoneally, and measurements were taken for sleep latency and duration. ICR mice (*n* = 8) used in the experiment had an adaptation period of one week before the experiment. Mice were segregated randomly into four groups: pentobarbital-treated group; pentobarbital + Zizy (200 mg/kg)-treated group; pentobarbital + caffeine-treated group; and pentobarbital + Zizy (200 mg/kg) + caffeine-treated group.

### 2.6. Electroencephalography (EEG) and Electromyogram (EMG) Recording Schedule

After a one-week adaptation period, SD rats underwent EEG and EMG implantation, followed by an additional one-week recovery after surgery. For EEG recordings, rats were divided into three groups for each experiment: control (saline), Zizy at doses of 100 mg/kg, and 200 mg/kg (*n* = 6). All treatments were administered orally for one week. EEG/EMG recordings were performed twice, one day before treatment initiation and one hour after the final treatment, as detailed in a previous study. The initial EEG recording served to identify any defects from the electrode implantation surgery and to ensure stable EEG transmission. Subsequently, the final EEG measurement was utilized for analysis. This last EEG recording was conducted under conditions in which the experimental apparatus accommodated each rat for three days before the recording took place to have the rats get acclimated to the tethered environment. After the completion of the procedures, the rats were sacrificed, and their brains were collected for further analysis.

### 2.7. Implantation Surgery for EEG and EMG

Under inhaled isoflurane anesthesia (2–5% in O_2_), all surgical procedures were conducted in a dedicated stereotaxic frame. Rats (*n* = 6) underwent implantation of EEG and EMG electrodes, following previously established methods [22,23,24]. Initially, the head was shaved and disinfected with alcohol and betadine povidone-iodine. To help the rats survive, 2 mL of sterile saline and 5% glucose were administered intraperitoneally. A 1 cm incision was made in the midline of the scalp using a surgical scalpel blade and surgical scissors. The periosteum was then cleaned with a sterile cotton swab, and lidocaine HCl injection (2%), a local anesthetic, was appropriately applied to the skull. The skull was aligned using bregma and lambda as reference points, and then microscopic holes were carefully drilled to match the established coordinates for electrode insertion. A schematic representation of the EEG/EMG coordinates is presented in Figure 1B. The surgical procedure involved placing a reference ground screw in the frontal area, bilateral EEG screws on the cerebral cortex, and two depth electrodes—one in the left thalamus (coordinates from bregma: AP = −3.0 mm, ML = −3.0 mm, DV = −6.4 mm) and the other in the right hippocampus (coordinates from bregma: AP = −2.8 mm, ML = +1.8 mm, DV = −3.3 mm). Furthermore, a pair of EMG electrodes, crafted from 15 mm nylon-insulated silver wire (0.013” coated, A-M Systems, Sequim, WA, USA), were stitched into the superior nuchal muscles situated at the back of the neck (Figure 1B). The seven EEG/EMG leads were soldered to female contacts (E363/0; P1 Technologies, Roanoke, VA, USA) and placed within a 7-channel pedestal (MS7Pl; P1 Technologies). The base of the pedestal and screw heads were secured with dental acrylic (self-curing set; Vertex Dental BV, Zeist, The Netherlands), and the skin was sutured, leaving only the pedestal socket exposed. Rats were administered post-operative analgesia (ketoprofen, 2.5 mg/kg, i.p.) and individually housed in cages to recover for one week before recording sessions commenced.

### 2.8. EEG and EMG Recording

To record EEGs and EMGs, a 7-pin EEG/EMG cable (363–363; P1 Technologies) was inserted into the implanted pedestal and then connected to a commutator. EEG/EMG signals were gathered and stored without interruption at 200 Hz sampling rates through a recording amplifier (AURA24; Grass-Telefactor, West Warwick, RI, USA) and data acquisition software (Twin 4.5.3; Grass Technologies, West Warwick, RI, USA). The extracted files were applied with a notch filter to remove 60 Hz noise generated from transmission lines.

### 2.9. Automated Sleep–Wake State Scored Algorithm Using MATLAB

The software AccuSleep Version X, an open-source program [25] coded in MATLAB (R2021b, The MathWorks Inc., Natick, MA, USA) from GitHub (https://github.com/zekebarger/AccuSleep, 4 December 2024), semi-automatically scored the sleep–wake states (wakefulness, REMS, and NREMS) based on 5 s EEG/EMG signal epochs. In light of the circadian rhythm of rodents, 6 h (10:00–16:00) were exported and analyzed.

### 2.10. Time-Dependent Power Spectrum Heatmaps

Time–frequency power spectrum density (PSD), indicating EEG signal strength, was computed using short-time Fourier transform within a 0–20 Hz range. This analysis employed a 5 s window with a 0.25 s step. The PSD values and the percentage power density are presented across three distinct frequency bands, namely delta (0.5–4 Hz), theta (4–8 Hz), and alpha (8–13 Hz). The power density percentage was calculated and graphed under the assumption that the sum of the power densities of the three frequencies for each state equaled 1. The overall PSD of the EEG data in the 1–20 Hz frequency range, including delta, theta, and alpha, was also calculated.

### 2.11. Western Blot

After conducting the behavior test and EEG recording, selected brain samples (frontal cortex, hippocampus, and hypothalamus) were gathered. The samples were lysed with RIPA buffer from ELPIS Biotech Inc. (Daejeon, Republic of Korea). The protein samples were denatured and separated based on size using 12% SDS-PAGE, ensuring consistent migration for subsequent analysis. Following gel transfer onto a PVDF membrane, non-specific binding sites were blocked with 5% skim milk. Primary antibodies (1:1000), specific to the target protein, were then incubated with the membrane. Subsequent application of secondary antibodies yielded detectable signals through enhanced chemiluminescence (ECL, Bio-Rad, Hercules, CA, USA).

### 2.12. Intracellular Chloride Ion Measurement Assay

Using the SH-SY5Y human neuroblastoma cell line obtained from the Korean Cell Line Bank (Seoul, Republic of Korea), relative changes were assessed by employing the MQAE, a Cl^−^-sensitive indicator, based on established research [26,27]. Cultured cells at a concentration of 4 × 10^5^ cells/mL were exposed to 5 mM MQAE in Cl^−^-free buffer within a 96-well plate for 3 h. Following this, the cells underwent five washes and were then treated with either 500 µM bicuculline or 12.5 µM flumazenil for 15 min at room temperature. The fluorescent measurements were obtained from the plate using a fluorescent reader (Synergy H1, BioTek Instruments Inc., Winooski, VT, USA) at an extinction of 365 nm and emission of 450 nm.

### 2.13. Statistical Analysis

Graph Prism 5 software (GraphPad Software Inc. in San Diego, CA, USA) facilitated the statistical analysis. The data underwent scrutiny via one-way analysis of variance (ANOVA) using Tukey’s post hoc test with statistical significance acknowledged for *p*-values < 0.05.

## 3. Results

### 3.1. Administration of Zizyphi Semen Extract and Jujuboside A Prolongs Sleep Duration in Pentobarbital-Induced Sleep in Mice

**Figure 2:**
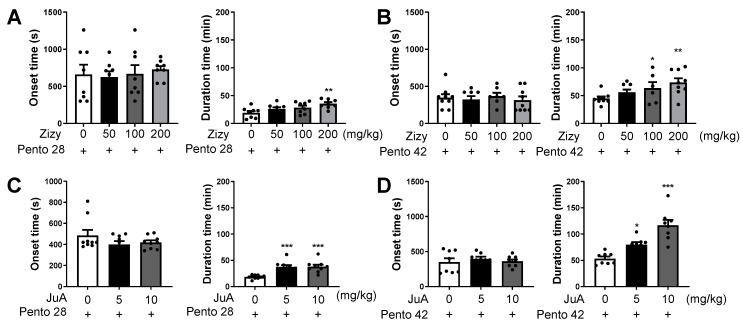
Effects of Zizy extract and JuA on sleep onset and sleep duration in pentobarbital-treated mice. The effect of Zizy extract (**A**,**B**) and JuA (**C**,**D**) on sleep onset and sleep duration was evaluated at subhypnotic (**A**) and hypnotic (**B**) concentrations of pentobarbital. The mice were orally administered Zizy extract at each concentration (50, 100, 200 mg/kg) or intraperitoneally administered JuA at each concentration (5, 10 mg/kg), 1 h and 25 h before the intraperitoneal administration of pentobarbital. Mice were fasted during the 24 h interval between Zizy extract administration. Data are expressed as the mean ± SEM (*n* = 8–9) and were analyzed by one-way ANOVA followed by Tukey’s post hoc test. * *p* < 0.05, ** *p* < 0.01, and *** *p* < 0.001 compared with the pentobarbital only group. Zizy, Zizyphi Semen extract; JuA, jujuboside A; Pento, pentobarbital.

The sleep-enhancing effects of Zizyphi Semen (Zizy) extract and its bioactive compound, jujuboside A (JuA), were investigated using a previously established pentobarbital-induced sleep mice model under various conditions. Notably, sleep duration exhibited a dose-dependent augmentation under the influence of pentobarbital. Specifically, administering pentobarbital alone led to an average sleep duration of 19.1 min at a dosage of 28 mg/kg and an average of 44.9 min at 42 mg/kg. Intriguingly, pretreatment with Zizy extract resulted in a sizable increase in sleep duration, up to 45 min, in mice subjected to the subhypnotic dosage of 28 mg/kg pentobarbital (Figure 2A). Similarly, mice receiving the hypnotic dosage of 42 mg/kg pentobarbital and pretreated with Zizy extract demonstrated a dose-dependent elevation in sleep duration (Figure 2B). Notably, sleep onset remained unaltered in all groups, thereby underscoring the specificity of the Zizy extract effect on prolonging sleep duration without affecting the time to initiate sleep. The investigation explored the role of JuA, a prominent bioactive constituent of Zizy, in sleep experiments utilizing both 28 mg/kg and 42 mg/kg pentobarbital dosages. Remarkably, JuA exhibited a consistent increase in sleep duration in mice (Figure 2C), aligning with the effects observed with Zizy extract pretreatment (Figure 2D). Moreover, a noteworthy reduction in sleep onset time was observed with JuA administration, albeit not reaching statistical significance, in both pentobarbital dosages. These findings highlight the sleep-promoting properties of JuA and demonstrate its contribution to the overall efficacy of Zizy extract in enhancing sleep duration.

### 3.2. Administration of Zizyphi Semen Extract Relieves Caffeine-Induced Insomnia in Mice

**Figure 3:**
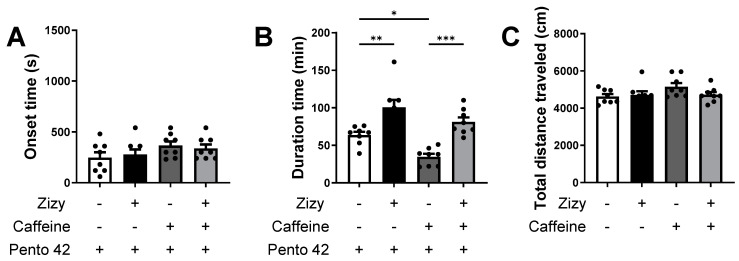
Effects of Zizy extract on sleep onset, sleep duration, and locomotion in mice with caffeine-induced insomnia. Mice with caffeine-induced insomnia were subjected to a pentobarbital-induced sleep mouse model, and sleep onset (**A**) and sleep duration (**B**) were measured. The motility (**C**) of the mice was measured for 20 min through the OFT, 30 min before pentobarbital injection. The Zizy extract (200 mg/kg) was orally administered twice, 24 h apart. Then, 20 min after the last administration of Zizy extract, caffeine (10 mg/kg) or saline was intraperitoneally administered. OFT was performed for 20 min, before intraperitoneal administration of pentobarbital (42 mg/kg). Data are expressed as the mean ± SEM (*n* = 8) and were analyzed by one-way ANOVA followed by Tukey’s post hoc test. * *p* < 0.05, ** *p* < 0.01 and *** *p* < 0.001 compared with the exclusively pentobarbital-treated group. Zizy, Zizyphi Semen extract; Pento, pentobarbital.

The efficacy of Zizy extract on insomnia was explored using caffeine-induced insomniac mice followed by a pentobarbital-induced sleep model. When caffeine-induced insomnia decreased pentobarbital-induced sleep duration by 45.47%, with an average sleep time of 34.63 min compared to the control (63.5 min), Zizy extract oral administration remarkably amplified the reducing sleep duration by 134.3%, averaging 81.13 min (Figure 3A,B). This signified an alleviation of caffeine-induced insomnia by Zizy extract. Prior to pentobarbital injection, the locomotor activity of OFT in mice was affected by caffeine and Zizy extract (Figure 3C). While caffeine seemed to increase activity levels, Zizy extract administration did not show significant changes. These findings strongly support that Zizy extract has an effect against caffeine-induced insomnia without influencing wake-state locomotion.

### 3.3. Administration of Zizyphi Semen Extract Modulates Sleep Architecture in Rats

**Figure 4:**
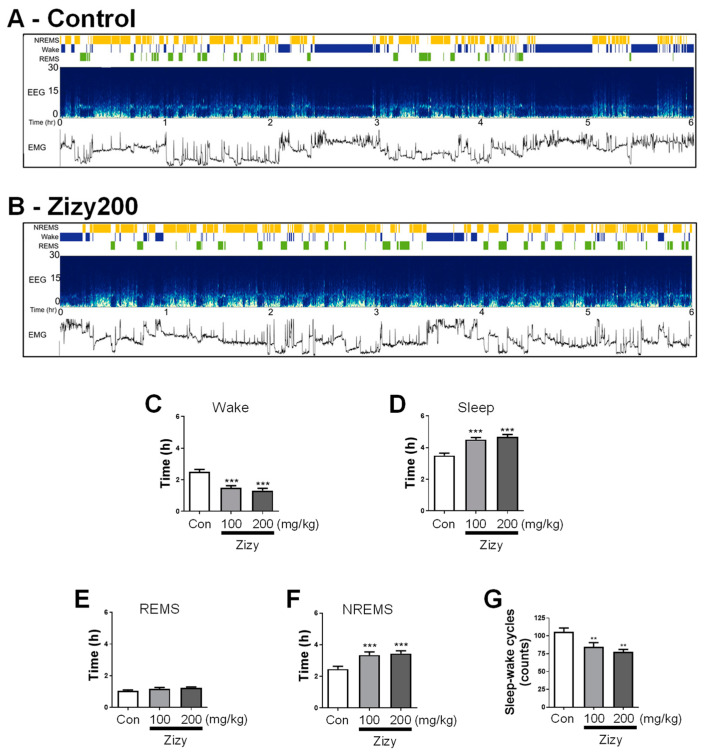
Effects of Zizy extract on sleep architecture in rats analyzed by the automatic sleep scoring algorithm. The representative instances of the AccuSleep interface, exhibiting sleep stages, EEG spectrograms, and EMG power in both the control and Zizy200, are shown (**A**,**B**). The analysis encompassed wake and sleep times (**C**,**D**), REMS and NREMS durations (**E**,**F**), as well as sleep–wake cycles (**G**). The sleep assessment employed the AccuSleep algorithm, programmed in MATLAB, for automated sleep scoring. For seven days, Zizy extract was orally administered at doses of 100, 200 mg/kg once daily. Following the last administration, EEG and EMG recordings were carried out, focusing on a 6 h window (10:00–16:00) for analysis. The analysis was divided into each sleep state using a 5 s epoch via an automatic sleep scoring algorithm. Data are expressed as the mean ± SEM (*n* = 6) and were analyzed by one-way ANOVA followed by Tukey’s post hoc test. ** *p* < 0.01 and *** *p* < 0.001 compared with the control group. Zizy, Zizyphi Semen extract; EEG, electroencephalogram; EMG, electromyogram; REMS, rapid eye movement sleep; NREMS, non-REMS; Wake, wakefulness.

The effect of Zizy extract on sleep architecture was assessed through EEG/EMG recordings in rats with implanted electrodes. AccuSleep, an automatic sleep scoring algorithm written in MATLAB, displayed the results of sleep structural analysis for each subject in the interface shown in Figure 4A,B. A representative interface from each group contained continuous EEG/EMG patterns over a 6 h period (10:00–16:00). The group orally administered Zizy extract (100, 200 mg/kg) for 7 days showed reduced wake time and greater total sleep time in comparison to the control group (Figure 4C,D). Sleep duration was further classified into REMS and NREMS through EEG analysis. Although the REMS distribution was unchanged (Figure 4E), the NREMS period was noticeably prolonged (Figure 4F). In particular, administration of Zizy extract significantly reduced the sleep–wake cycle at both doses (100, 200 mg/kg) (Figure 4G). These results strongly suggest that Zizy extract is likely to primarily affect sleep maintenance by contributing to the prolongation of sleep duration, particularly NREMS, and alleviating insomnia.

### 3.4. Administration of Zizyphi Semen Extract Tends to Modulate the Power Density of EEG Waves During REMS in Rats

**Figure 5:**
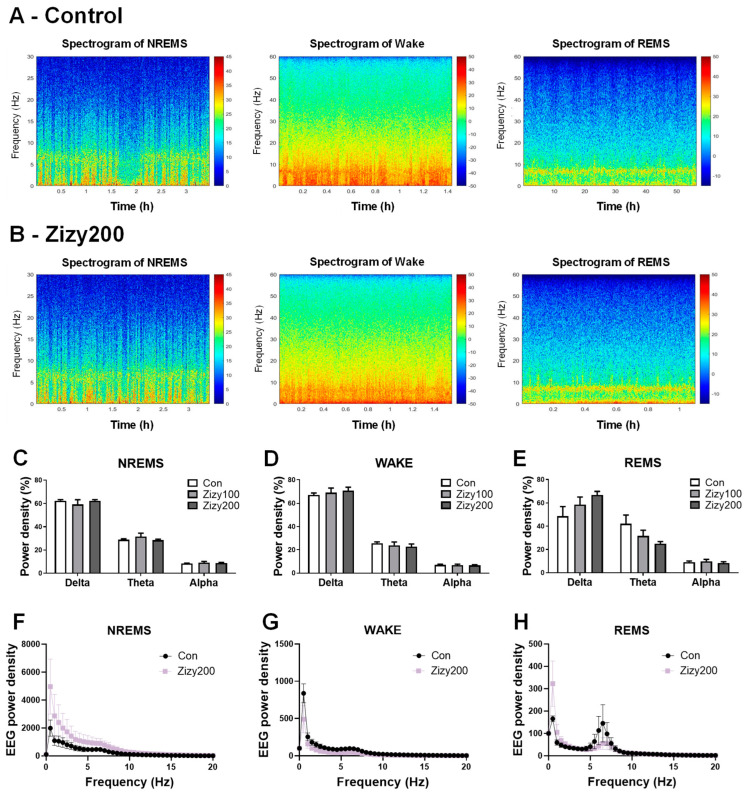
Effects of Zizy extract on sleep structure, shown as time-dependent power spectral heatmaps and power densities of specific wavelengths. The power spectrum heatmaps illustrate the distinctive features of each group during the REMS, Wake, and NREMS states (**A**,**B**), with time indicated on the x-axes and frequency on the y-axes. A Jet colormap was utilized to represent the data range depicted on the right side of the heatmap. The % of EEG power spectrum densities during NREMS (**C**), Wake (**D**), and REMS (**E**). Spectral distributions of EEG power density in the frequencies between 0.5 and 20 Hz in NREMS (**F**), Wake (**G**), and REMS (**H**). Zizy extract (100, 200 mg/kg, p.o.) was given once daily for 7 days. After the final administration, EEG and EMG recordings were conducted, and a 6 h period (10:00–16:00) was extracted and used for analysis. Data are expressed as the mean ± SEM (*n* = 6) and were analyzed by one-way ANOVA followed by Tukey’s post hoc test. Zizy, Zizyphi Semen extract; REMS, rapid eye movement sleep; NREMS, non-REMS; Wake, wakefulness.

Using power spectrogram analysis, the effect of Zizy extract on the power density of distinct wavelengths was assessed. Representative time-dependent power spectral heatmaps provided a comprehensive overview of wakefulness, REMS, and NREMS states across both the control and Zizy200 groups (Figure 5A,B). Expression range adjustments were made to distinctly display the characteristics of each state. Further quantitative analysis (Figure 5C–E) indicated that Zizy extract administration for 7 days had no observable effect on the delta (δ), theta (θ), and alpha (α) power densities during NREMS and WAKE. In REMS, there appeared to be a trend towards an increased delta (δ) and a decreased theta (θ), although these changes did not reach statistical significance. The results of graphing the overall power density level in the range of 0–20 Hz including the delta, theta, and alpha also showed changing power density levels in REMS, but no significant differences were found (Figure 5F–H). These results suggest that Zizy extract did not significantly alter the power density of specific wavelengths in each sleep state.

### 3.5. Administration of Zizyphi Semen Extract Modulates the Expression of GABA_A_ Receptor Subunits and GAD65/67 in the Rat Brain

**Figure 6:**
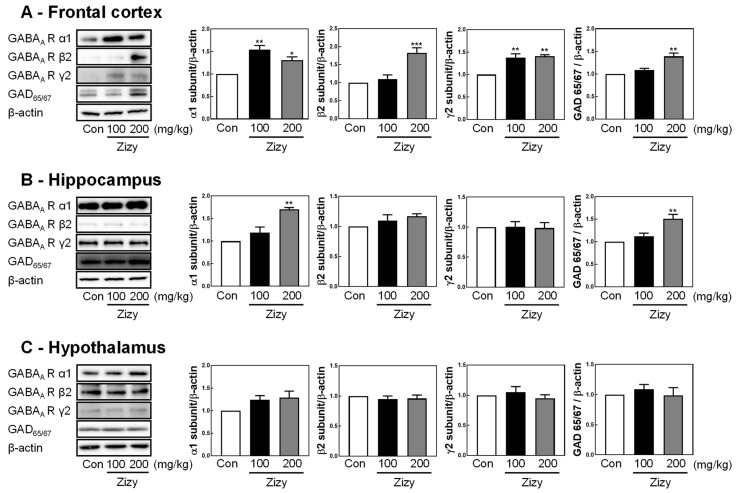
Effects of Zizy extract on the modulation of GAD65/67 and GABA_A_ receptor subunits. Western blot analysis of (**A**) rat frontal cortex, (**B**) hippocampus, and (**C**) hypothalamus after EEG analysis. The presented findings represent the results from three separate experiments, with quantification data provided in the right panel. Data are expressed as the mean ± SEM (*n* = 3) and were analyzed by one-way ANOVA followed by Tukey’s post hoc test. * *p* < 0.05, ** *p* < 0.01, and *** *p* < 0.001 compared with the control group. Zizy, Zizyphi Semen extract.

Following EEG recordings, the impact of Zizy extract on GABA_A_ receptor subtypes and GAD65/67 in the rat brain was investigated to evaluate its potential mechanisms for sleep-prolonging effects. In the frontal cortex, Zizy extract treatment notably increased the expression of α1, β2, and γ2 subunits and GAD65/67 proteins (Figure 6A). Similarly, in the hippocampus, the administration of a high concentration of Zizy extract (200 mg/kg) enhanced the protein expression of the α1 subunit and GAD65/67 (Figure 6B). Meanwhile, the hypothalamus exhibited no discernible alterations in the protein expression levels of different GABA_A_ receptor subtypes following Zizy extract treatment (Figure 6C). These findings highlight the region-specific effects of Zizy extract on GABA_A_ receptor subtypes, suggesting its potential as a therapeutic candidate for sleep disorders by targeting specific brain regions.

### 3.6. Jujuboside A Induces Intracellular Chloride Influx in SH-SY5Y Cells

**Figure 7:**
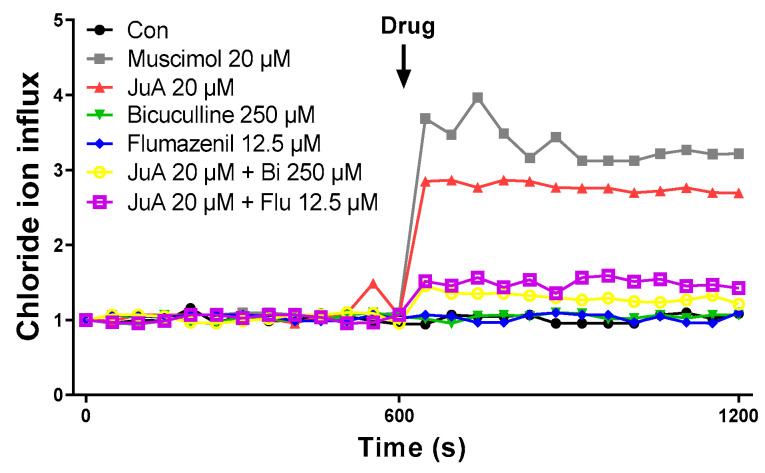
Effect of jujuboside A on Cl^−^ influx in human neuroblastoma SH-SY5Y cells. Intracellular chloride influx was detected by *N*-(ethoxycarbonylmethyl)-6-methoxyquinolinium bromide (MQAE). Cells were exposed to 5 mM MQAE for 3 h, followed by 15 min of treatment with 500 µM bicuculline or 12.5 µM flumazenil. Fluorescence was observed through excitation at 365 nm and emission at 450 nm, measuring intracellular chloride influx via fluorescence quenching. JuA, jujuboside A; Bi, bicuculline; Flu, flumazenil.

Displayed in Figure 7 is the effect of JuA, a characterizing constituent of Zizy extract, on intracellular Cl^−^ influx in vitro using the human SH-SY5Y neuroblastoma cell line. Treatment with JuA led to a rise in intracellular chloride influx, similar to the effect seen with muscimol. The concentration of JuA used was determined through cell viability and cytotoxicity assays, referencing previous studies. Bicuculline, acting as a competitive GABA_A_ receptor antagonist, and flumazenil, a benzodiazepine receptor antagonist, were included in this investigation. Muscimol served as a positive control, exerting itself as a potent GABA_A_ receptor agonist. Pretreatment with the individual antagonist itself did not produce significant changes in MQAE fluorescence values. However, when flumazenil or bicuculline was applied before JuA, intracellular Cl^−^ influx was noticeably reduced compared to when JuA was applied alone. These findings suggest that JuA stimulates Cl^−^ influx by modulating GABA_A_ receptors.

## 4. Discussion

Adequate sleep not only promotes memory consolidation and enhancement in learning, but also regulates mood and emotional responses, reducing the risk of mental health disorders such as depression, anxiety, and mood disturbances [9,28]. Although the etiology of sleep disorders is not thoroughly understood, salient features of insomnia are difficulty in falling asleep and maintaining and waking up from sleep. While numerous drugs aim to alleviate insomnia, their use frequently leads to severe side effects [10]. By the same token, there is a notable rise in the production of alternative supplements from natural sources with attenuated side effects. Zizyphi Semen (Zizy) has been traditionally consumed in the Orient as tea to aid in sleep. Zizy also contains jujuboside A (JuA) as a dammarane-type saponin [14]. Although Zizy has been acknowledged for its calming properties, explanations regarding its primary soothing compounds and mechanism, particularly concerning the results of electroencephalogram (EEG) and electromyogram (EMG), are still limited. This study aimed to investigate whether the effect of prolonged sleep duration of Zizy extract (ext) was related to improvements in sleep quality and to provide insights into its neurological mechanisms.

The pentobarbital-induced sleep mouse model is a well-established approach for evaluating hypnotic substances [29,30]. In the present study, Zizy extract and its bioactive constituent, jujuboside A, both effectively enhanced pentobarbital-induced sleep duration without changing sleep onset time, which suggests JuA’s pivotal role in prolonging sleep duration by Zizy extract. Additionally, in a caffeine-induced insomniac mouse model, administration of Zizy extract improved insomnia without affecting locomotor activity. Zizy extract potentiated the effects of pentobarbital, which acts through GABA_A_ receptor activation, suggesting that the CNS depressant effect of Zizy extract was probably mediated through chloride channel opening. Consequently, these findings suggest Zizy extract to be a promising candidate for extending sleep duration, offering an alternative approach to traditional hypnotics, with a focus on natural products and reduced side effects.

Sleep is a complex phenomenon encompassing two major stages: non-rapid eye movement sleep (NREMS) and rapid eye movement sleep (REMS) [18]. These sleep stages can be effectively studied and quantified through electroencephalogram (EEG) and electromyogram (EMG) analyses, which recorded neural electrical activity and muscle responses, respectively [31,32]. Utilizing a pentobarbital-induced sleep model, the present researchers established a dose–response relationship for Zizy extract and conducted EEG/EMG analysis after identifying an effective dose. Notably, Zizy extract administration led to a decrease in wakefulness duration and an increase in overall sleep duration, corroborating the results of pentobarbital-induced sleep tests. Furthermore, Zizy extract effectively reduced the frequency of awakenings during sleep, suggesting an improvement in sleep quality. To further measure sleep quality in rodents, we analyzed brain wave changes and intensity across sleep structures using delta, alpha, and theta activity. Zizy extract tended to enhance delta activity during REMS but did not affect NREM or WAKE state power density. It is expected that significant changes will be observed if the intake concentration and period are more adjusted. In conclusion, Zizy extract not only prolonged sleep time but also increased NREMS time, indicating the potential to induce brain wave changes.

Sleep–wake cycle regulation involves the interplay of various neurotransmitters, including GABA, serotonin, adenosine, and histamine [33,34]. Considering that pentobarbital-induced sleep is promoted through the GABAergic system [35,36], this paper investigated whether the sleep-prolonging effect of Zizy extract pretreatment was mediated by effects on the central nervous system (CNS) through the GABAergic system. Focusing on key brain regions such as the hippocampus, frontal cortex, and hypothalamus, which play pivotal roles in memory consolidation, anxiety-related behaviors, and arousal regulation [37,38], we analyzed the protein expression levels of GABA_A_ receptor subunits and GAD65/67, an enzyme involved in GABA synthesis. Zizy extract exhibited notable modulation of the GAD65/67, alpha1, beta2, and gamma2 subunit in the frontal cortex and alpha1 and GAD65/67 in the hippocampus. However, such changes were not observed in the hypothalamus. These results suggest that the administration of Zizy extract can affect sleep by augmenting the expression of GABA receptors and neurons that affect sleep-related brain regions. GABA_A_ receptors are complex assemblies of subunits and function as chloride ion channels gated by GABA, benzodiazepine, and barbiturate ligands. Upon ligand binding, they allow a net influx of negative chloride ions, hyperpolarizing the neuronal membrane and diminishing neuronal firing, thus promoting sleep. Studies have demonstrated that drugs and GABA_A_ receptor agonists can enhance chloride uptake in GABA_A_ receptors [39]. In a human SH-SY5Y neuroblastoma cell line, JuA, a constituent of Zizy ext, increased chloride influx like a GABA_A_ receptor agonist, and this effect was not inhibited by bicuculline and flumazenil. Jujuboside A may act on the other type of chlorine influx channel, not the GABA receptor. Alternatively, it might not be ruled out that it acts by promoting the release of GABA in the cells.

## 5. Conclusions

This investigation holds the potential of offering valuable insights into the functions and underlying mechanisms of Zizy extract and its bioactive component, JuA, in the context of enhancing sleep duration. In contrast to conventional pharmacological interventions for sleep disorders fraught with undesirable side effects, Zizy ext, derived from natural sources, prolongs sleep duration and improves sleep quality in rodent models. Acknowledging the pivotal role of nutrition-based approaches in managing sleep disturbances, this research highlights the potential of Zizy extract as a dietary supplement for addressing sleep disorders.

## Data Availability

The original contributions presented in this study are included in the article. Further inquiries can be directed to the corresponding author. The data are not publicly available due to privacy.
